# Towards a scientific concept of free will as a biological trait: spontaneous actions and decision-making in invertebrates

**DOI:** 10.1098/rspb.2010.2325

**Published:** 2010-12-15

**Authors:** Björn Brembs

**Affiliations:** Freie Universität Berlin, Institute for Biology – Neurobiology, Königin-Luise-Strasse 28/30, 14195 Berlin, Germany

**Keywords:** invertebrate, spontaneous, volition, action

## Abstract

Until the advent of modern neuroscience, free will used to be a theological and a metaphysical concept, debated with little reference to brain function. Today, with ever increasing understanding of neurons, circuits and cognition, this concept has become outdated and any metaphysical account of free will is rightfully rejected. The consequence is not, however, that we become mindless automata responding predictably to external stimuli. On the contrary, accumulating evidence also from brains much smaller than ours points towards a general organization of brain function that incorporates flexible decision-making on the basis of complex computations negotiating internal and external processing. The adaptive value of such an organization consists of being unpredictable for competitors, prey or predators, as well as being able to explore the hidden resource deterministic automats would never find. At the same time, this organization allows all animals to respond efficiently with tried-and-tested behaviours to predictable and reliable stimuli. As has been the case so many times in the history of neuroscience, invertebrate model systems are spearheading these research efforts. This comparatively recent evidence indicates that one common ability of most if not all brains is to choose among different behavioural options even in the absence of differences in the environment and perform genuinely novel acts. Therefore, it seems a reasonable effort for any neurobiologist to join and support a rather illustrious list of scholars who are trying to wrestle the term ‘free will’ from its metaphysical ancestry. The goal is to arrive at a scientific concept of free will, starting from these recently discovered processes with a strong emphasis on the neurobiological mechanisms underlying them.

## 1. Introduction: the rejection of the metaphysical concept of free will

What could possibly get a neurobiologist with no formal training in philosophy beyond a few introductory lectures, to publicly voice his opinion on free will? Even worse, why use empirical, neurobiological evidence mainly from invertebrates to make the case? Surely, the lowly worm, snail or fly cannot even be close to something as philosophical as free will? The main reason is this neurobiologist's opinion that free will is a biological trait and not a metaphysical entity. ‘Free will is a biological property, not a gift or a mystery’ [1]. Today, neurobiology has accumulated sufficient evidence that we can move on from speculating about the existence of free will towards plausible models of how brains have implemented it. On the surface, this statement seems to contradict public statements from many other neurobiologists who fervently deny free will. In fact, it appears that if neurobiologists feel compelled to write about free will, they do so only to declare that it is an illusion [2–5]. Of course, all of these neurobiologists are correct in that free will as a metaphysical entity indeed most probably is an illusion. Colloquial and historical use of the term ‘free will’ has been inextricably linked with one variant or another of dualism. There have been so many and thorough recounts of the free will debate, that I will only reference some, which can serve to introduce the concepts used here [6–9]. Psychologists and neurobiologists have rightfully pointed out for decades now that there is no empirical support for any form of dualism. The interactionism proposed by Popper & Eccles was probably one of the last prominent accounts of dualism [10]. Since then, these and related positions have largely fallen into irrelevance. Today, the metaphysical concept of free will is largely devoid of any support, empirical or intellectual.

## 2. The rejection of determinism

That said, it is an all too common misconception that the failure of dualism as a valid hypothesis automatically entails that brains are deterministic and all our actions are direct consequences of gene–environment interactions, maybe with some random stochasticity added in here and there for good measure [2]. It is tempting to speculate that most, if not all, scholars declaring free will an illusion share this concept. However, our world is not deterministic, not even the macroscopic world. Quantum mechanics provides objective chance as a trace element of reality. In a very clear description of how keenly aware physicists are that Heisenberg's uncertainty principle indeed describes a property of our world rather than a failure of scientists to accurately measure it, Stephen Hawking has postulated that black holes emit the radiation named after him [11], a phenomenon based on the well-known formation of virtual particle–antiparticle pairs in the vacuum of space. The process thought to underlie Hawking radiation has recently been observed in a laboratory analogue of the event horizon [12,13]. On the ‘mesoscopic’ scale, fullerenes have famously shown interference in a double-slit experiment [14]. Quantum effects have repeatedly been observed directly on the nano-scale [15,16], and superconductivity (e.g. [17]) or Bose–Einstein condensates (e.g. [18]) are well-known phenomena. Quantum events such as radioactive decay or uncertainty in the photoelectric effect are used to create random-number generators for cryptography that cannot be broken into. Thus, quantum effects are being observed also on the macroscopic scale. Therefore, determinism can be rejected with at least as much empirical evidence and intellectual rigor as the metaphysical account of free will. ‘The universe has an irreducibly random character. If it is a clockwork, its cogs, springs, and levers are not Swiss-made; they do not follow a predetermined path. Physical indeterminism rules in the world of the very small as well as in the world of the very large’ [9].

## 3. Behavioural variability as an adaptive trait

If dualism is not an option and determinism is equally untenable, what other options are we left with? Some scholars have resorted to quantum uncertainty in the brain as the solution, providing the necessary discontinuity in the causal chain of events. This is not unrealistic, as there is evidence that biological organisms can evolve to take advantage of quantum effects. For instance, plants use quantum coherence when harvesting light in their photosynthetic complexes [19–22]. Until now, however, it has proved difficult to find direct empirical evidence in support of analogous phenomena in brains [9]. Moreover, and more importantly, the pure chance of quantum indeterminism alone is not what anyone would call ‘freedom’. ‘For surely my actions should be caused because I want them to happen for one or more reasons rather that they happen by chance’ [9]. This is precisely where the biological mechanisms underlying the generation of behavioural variability can provide a viable concept of free will.

Biologists need not resort to quantum mechanics to understand that deterministic behaviour can never be evolutionarily stable. Evolution is a competitive business and predictability is one thing that will make sure that a competitor will be out of business soon. There are many illuminating examples of selection pressures favouring unpredictability, but three recently published reports dealing with one of the most repeatable and hence best-studied class of behaviours are especially telling. These examples concern escape behaviours.

One of the most well-studied escape behaviours is the so-called C-start response in fishes. The response is called C-start because fishes that perceive sudden pressure changes on one side of their body bend in a C-shape away from the perceived stimulus to escape in the opposite direction. One of the largest neurons in vertebrate nervous systems is mediating this response, the Mauthner cell (e.g. [23]). Recently, Kenneth Catania and colleagues described the hunting technique of tentacled snakes (*Erpeton tentaculatus*) [24,25]. The snakes hunt for fishes by cunningly eliciting a C-start response in the potential prey animal with a more caudal part of their body, prompting the fish to C-start exactly into the mouth of the snake.

Some of the most important predators of earthworms are moles. When moles dig through the ground, they produce a very distinctive sound. Earthworms have evolved to respond to this sound by crawling to the surface, where the moles will not follow them. Kenneth Catania recently reported that the technique of ‘worm-grunting’, employed in order to catch earthworms as fish bait, exploits this response. The worm grunters use a combination of wooden poles and metal rods to generate the sound and then collect the worms from the surface [26].

In the third example, another very well-studied escape response is exploited by birds. Under most circumstances, the highly sophisticated jump response of dipteran flies is perfectly sufficient to catapult the animals out of harm's way (e.g. [27]). However, painted redstarts (*Myioborus pictus*) are ground-hunting birds that flush out dipterans by eliciting their jump response with dedicated display behaviours. Once the otherwise well-camouflaged flies have jumped, they are highly visible against the bright sky and can be caught by the birds [28,29].

It is not a huge leap to generalize these insights from escape responses to other behaviours. Predictability can never be an evolutionarily stable strategy. Instead, animals need to balance the effectiveness and efficiency of their behaviours with just enough variability to spare them from being predictable. Efficient responses are controlled by the environment and thus vulnerable. Conversely, endogenously controlled variability reduces efficiency but increases vital unpredictability. Thus, in order to survive, every animal has to solve this dilemma. It is no coincidence that ecologists are very familiar with a prominent, analogous situation, the exploration/exploitation dilemma (originally formulated by March [30]): every animal, every species continuously faces the choice between staying and efficiently exploiting a well-known, but finite resource and leaving to find a new, undiscovered, potentially much richer, but uncertain resource. Efficiency (or optimality) always has to be traded off with flexibility in evolution, on many, if not all, levels of organization.

A great invertebrate example of the sort of Protean behaviour [31,32] selected for by these trade-offs is yet another escape behaviour, that of cockroaches. The cerci of these insects have evolved to detect minute air movements. Once perceived, these air movements trigger an escape response in the cockroach away from the side where the movement was detected. However, which angle with respect to the air movement is taken by the animal cannot be predicted precisely, because this component of the response is highly variable [33]. Therefore, in contrast to the three examples above, it is impossible for a predator to predict the trajectory of the escaping animal.

## 4. Brains are in control of variability

Competitive success and evolutionary fitness of all ambulatory organisms depend critically on intact behavioural variability as an adaptive function [34]. Behavioural variability is an adaptive trait and not ‘noise’. Not only biologists are aware of the fitness advantages provided by unpredictable behaviour, but philosophers also realized the adaptive advantages of behavioural variability and their potential to serve as a model for a scientific account of free will, as long as 25 years ago (e.g. [6]). The ultimate causes of behavioural variability are thus well understood. The proximate causes, however, are much less studied. One of the few known properties is that the level of variability also can vary. Faced with novel situations, humans and most animals spontaneously increase their behavioural variability [35–38]. Animals even vary their behaviour when a more stereotyped behaviour would be more efficient [39].

These observations suggest that there must be mechanisms by which brains control the variability they inject into their motor output. Some components of these mechanisms have been studied. For instance, tethered flies can be trained to reduce the range of the variability in their turning manoeuvres [40]. For example, one such stationary flying fly may be trained to cease generating left-turning manoeuvres by heating the fly (with an infrared heat beam) whenever it initiates such actions and by not heating it during right-turning manoeuvres. Before such training, it would generate left- and right-turning manoeuvres in equal measure. Protein kinase C activity is required for such a reduction [41]. Interestingly, analogous to the exploration–exploitation dilemma mentioned above, the mechanism by which the animals learn to decrease their behavioural variability (‘self-learning’) interacts with the learning mechanism by which the animals learn about external stimuli (‘world learning’). Part of this interaction balances self- and world learning such that self-learning (i.e. the endogenous reduction in behavioural variability) is slowed down whenever the world-learning mechanism is engaged. This part of the interaction is mediated by a subpopulation of neurons in a part of the insect brain called the mushroom bodies [42,43]. This population of neurons ensures that animals preferentially learn from their environment and reduce their endogenous behavioural variability only when there are good reasons for doing so. Such an organization may underlie the need for practice in order to reduce our behavioural variability when learning new skills, e.g. the basketball free-throw or the golf swing. The parallel to the exploration–exploitation dilemma lies in the balance between the endogenous and exogenous processing these interactions bestow upon the animal: learning about the world first allows the animal to keep its behaviour flexible in case the environment changes, while at the same time being able to efficiently solve the experimental task. If, however, it turns out that the environment does not change, then—and only then—is the circuitry controlling the behaviour itself modified, to more permanently alter the behaviour-generating process itself and thereby maximize on efficiency by reducing the endogenous variability.

Animals other than insects also learn to control their variability using feedback from the environment, such that levels of behavioural variability—from highly predictable to random-like—are directly influenced by reinforcement. For instance, consummatory feeding behaviour of the marine snail *Aplysia* is highly variable [44,45]. Recent evidence suggests that the seemingly rhythmic cycling of biting, swallowing and rejection movements of the animal's radula (a tongue-like organ) vary in order to be able to adapt to varying food sources [46]. In fact, much like the reduced variability in flies trained to avoid heat in the self-learning paradigm explained above, *Aplysia* can be trained to reduce the variability in their feeding behaviour and generate rhythmic, stereotyped movements [47–52]. It also takes practice for snails to become efficient and predictable. The default state is to behave variably and unpredictably.

The mechanisms to control behavioural variability are in place also in humans. For instance, depression and autism are characterized by abnormally stereotypic behaviours and a concomitant lack of behavioural variability. Patients suffering from such psychopathologies can learn to vary their behaviours when reinforced for doing so [53,54]. Also, the interactions between world- and self-learning seem to be present in vertebrates: extended training often leads to so-called habit formation, repetitive responses, controlled by environmental stimuli (e.g. [55,56]). It is intriguing that recent fMRI studies have discovered a so-called default-mode network in humans, the fluctuations in which can explain a large degree of the individual's behavioural variability [57], and that abnormalities in this default network are associated with most psychiatric disorders [58–60].

## 5. What are the neural mechanisms generating behavioural variability?

It thus appears that behavioural variability is a highly adaptive trait, under constant control of the brain balancing the need for variability with the need for efficiency. How do brains generate and control behavioural variability in this balance? These studies have only just begun. As was the case in much of neuroscience's history, be it ion channels, genes involved in learning and memory, electrical synapses or neurotransmitters, invertebrate model systems are leading the way in the study of the neural mechanisms underlying behavioural variability as well.

Two recent reports, concerned another highly reproducible (and therefore well-studied) behaviour, optomotor responses. Tethered flies respond to a moving grating in front of them with characteristic head movements in the same direction as the moving grating, aimed at stabilizing the image on the retina. By recording from motion-sensitive neurons in fly optic lobes, the authors found that the variability in these neurons did not suffice to explain the variability in the head movements [61,62]. Presumably, downstream neurons in the central brain inject additional variability, not present in the sensory input, which is reflected in the behaviour.

A corresponding conclusion can be drawn from two earlier studies, which independently found that the temporal structure of the variability in spontaneous turning manoeuvres both in tethered and in free-flying fruitflies could not be explained by random system noise [63,64]. Instead, a nonlinear signature was found, suggesting that fly brains operate at criticality, meaning that they are mathematically unstable, which, in turn, implies an evolved mechanism rendering brains highly susceptible to the smallest differences in initial conditions and amplifying them exponentially [63]. Put differently, fly brains have evolved to generate unpredictable turning manoeuvres. The default state also of flies is to behave variably. Ongoing studies are trying to localize the brain circuits giving rise to this nonlinear signature.

Results from studies in walking flies indicate that at least some component of variability in walking activity is under the control of a circuit in the so-called ellipsoid body, deep in the central brain [65]. The authors tested the temporal structure in spontaneous bouts of activity in flies walking back and forth individually in small tubes and found that the power law in their data disappeared if a subset of neurons in the ellipsoid body was experimentally silenced. Analogous experiments have recently been taken up independently by another group and the results are currently being evaluated [66]. The neurons of the ellipsoid body of the fly also exhibit spontaneous activity in live imaging experiments [67], suggesting a default-mode network also might exist in insects.

Even what is often presented to students as ‘the simplest behaviour’, the spinal stretch reflex in vertebrates, contains adaptive variability. Via the cortico-spinal tract, the motor cortex injects variability into this reflex arc, making it variable enough for operant self-learning [68–72]. Jonathan Wolpaw and colleagues can train mice, rats, monkeys and humans to produce reflex magnitudes either larger or smaller than a previously determined baseline precisely because much of the deviations from this baseline are not noise but variability deliberately injected into the reflex. Thus, while invertebrates lead the way in the biological study of behavioural variability, the principles discovered there can be found in vertebrates as well.

One of the common observations of behavioural variability in all animals seems to be that it is not entirely random, yet unpredictable. The principle thought to underlie this observation is nonlinearity. Nonlinear systems are characterized by sensitive dependence on initial conditions. This means such systems can amplify tiny disturbances such that the states of two initially almost identical nonlinear systems can diverge exponentially from each other. Because of this nonlinearity, it does not matter (and it is currently unknown) whether the ‘tiny disturbances’ are objectively random as in quantum randomness or whether they can be attributed to system, or thermal noise. What can be said is that principled, quantum randomness is always some part of the phenomenon, whether it is necessary or not, simply because quantum fluctuations do occur. Other than that it must be a non-zero contribution, there is currently insufficient data to quantify the contribution of such quantum randomness. In effect, such nonlinearity may be imagined as an amplification system in the brain that can either increase or decrease the variability in behaviour by exploiting small, random fluctuations as a source for generating large-scale variability. A general account of such amplification effects had already been formulated as early as in the 1930s [73]. Interestingly, a neuronal amplification process was recently observed directly in the barrel cortex of rodents, opening up the intriguing perspective of a physiological mechanism dedicated to generating neural (and by consequence behavioural) variability [74].

## 6. Determinism versus indeterminism is a false dichotomy

Together with Hume, most would probably subscribe to the notion that ‘tis impossible to admit of any medium betwixt chance and an absolute necessity’ [75]. For example, Steven Pinker (1997, p. 54) concurs that ‘A random event does not fit the concept of free will any more than a lawful one does, and could not serve as the long-sought locus of moral responsibility’ [76]. However, to consider chance and lawfulness as the two mutually exclusive sides of our reality is only one way to look at the issue. The unstable nonlinearity, which makes brains exquisitely sensitive to small perturbations, may be the behavioural correlate of amplification mechanisms such as those described for the barrel cortex [74]. This nonlinear signature eliminates the two alternatives, which both would run counter to free will, namely complete (or quantum) randomness and pure, Laplacian determinism. These represent opposite and extreme endpoints in discussions of brain functioning, which hamper the scientific discussion of free will. Instead, much like evolution itself, a scientific concept of free will comes to lie between chance and necessity, with mechanisms incorporating both randomness and lawfulness. The Humean dichotomy of chance and necessity is invalid for complex processes such as evolution or brain functioning. Such phenomena incorporate multiple components that are both lawful and indeterminate. This breakdown of the determinism/indeterminism dichotomy has long been appreciated in evolution and it is surprising to observe the lack of such an appreciation with regard to brain function among some thinkers of today (e.g. [2]). Stochasticity is not a nuisance, or a side effect of our reality. Evolution has shaped our brains to implement ‘stochasticity’ in a controlled way, injecting variability ‘at will’. Without such an implementation, we would not exist.

A scientific concept of free will cannot be a qualitative concept. The question is not any more ‘do we have free will?’; the questions is now: ‘how much free will do we have?’; ‘how much does this or that animal have?’. Free will becomes a quantitative trait.

## 7. Initiating activity: actions versus responses

Another concept that springs automatically from acknowledging behavioural variability as an adaptive trait is the concept of actions. In contrast to responses, actions are behaviours where it is either impossible to find an eliciting stimulus or where the latency and/or magnitude of the behaviour vary so widely, that the term ‘response’ becomes useless.

A long history of experiments on flies provides accumulating evidence that the behaviour of these animals is much more variable than it would need to be, given the variability in the neurons mediating the stimulus-response chain (reviewed in [77]). For instance, in the study of the temporal dynamics of turning behaviours in tethered flies referenced above [63], one situation recorded fly behaviour in constant stimulus conditions, i.e. nothing in the exquisitely controlled environment of the animals changed while the turning movements were recorded. Yet, the flies kept producing turning movements throughout the experiment as if there had been stimuli in their environment. Indeed, the temporal structure in these movements was qualitatively the same compared with when there were stimuli to be perceived. This observation is only one of many demonstrating the endogenous character of behavioural variability. Even though there was nothing in the environment prompting the animals to change their behaviour, they kept initiating turning manoeuvres in all directions. Clearly, each of these manoeuvres was a self-initiated, spontaneous action and not a response to some triggering, external stimulus.

In fact, such self-initiated actions are a necessary prerequisite for the kind of self-learning described above [41–43]. At the start of the experiment, the fly cannot know that it is its own turning manoeuvres that cause the switch from cold to hot and vice versa. To find out, the fly has to activate the behavioural modules it has available in this restrained situation and has to register whether one of them might have an influence on the punishing heat beam. There is no appropriate sensory stimulus from outside to elicit the respective behaviour. The fly must have a way to initiate its behaviours itself, in order to correlate these actions with the changes in the environment. Clearly, the brain is built such that under certain circumstances the items of the behavioural repertoire can get released independent of sensory stimuli.

The fly cannot know the solutions to most real-life problems. Beyond behaving unpredictably to evade predators or outcompete a competitor, all animals must explore, must try out different solutions to unforeseen problems. Without behaving variably, without acting rather than passively responding, there can be no success in evolution. Those individuals who have found the best balance between flexible actions and efficient responses are the ones who have succeeded in evolution. It is this potential to behave variably, to initiate actions independently of the stimulus situation, which provides animals with choices.

## 8. Freedom of choice

The neurobiological basis of decision-making can also be studied very well in invertebrate models. For instance, isolated leech nervous systems chose either a swimming motor programme or a crawling motor programme to an invariant electrical stimulus [78–80]. Every time the stimulus is applied, a set of neurons in the leech ganglia goes through a so far poorly understood process of decision-making to arrive either at a swimming or at a crawling behaviour. The stimulus situation could not be more perfectly controlled than in an isolated nervous system, excluding any possible spurious stimuli reaching sensory receptors unnoticed by the experimenter. In fact, even hypothetical ‘internal stimuli’, generated somehow by the animal must in this case be coming from the nervous system itself, rendering the concept of ‘stimulus’ in this respect rather useless. Yet, under these ‘carefully controlled experimental circumstances, the animal behaves as it damned well pleases’ (Harvard Law of Animal Behaviour) [34].

Seymour Benzer, one of the founders of Neurogenetics, captured this phenomenon in the description of his first phototaxis experiments in 1967: ‘ … if you put flies at one end of a tube and a light at the other end, the flies will run to the light. But I noticed that not every fly will run every time. If you separate the ones that ran or did not run and test them again, you find, again, the same percentage will run. But an individual fly will make its own decision’. (cited from Brown & Haglund (1994) *J. NIH Res.* **6**, 66–73). Not even 10 years later, Quinn *et al.* separated flies, conditioned to avoid one of two odours, into those that did avoid the odour and those that did not. In a subsequent second test, they found that both the avoiders and the non-avoiders separated along the same percentages as in the first test, prompting the authors to conclude: ‘This result suggests that the expression of learning is probabilistic in every fly’ [81]. Training shifted the initial 50–50 decision of the flies away from the punished odour, but the flies still made the decisions themselves—only with a different probability than before training. Most recently, in the experiments described above, the tethered flies without any feedback made spontaneous decisions to turn one way or another [63]. These are only three examples from more than 40 years in which many behavioural manifestations of decision-making in the fly brain have been observed. Like heat, flies can control also odour intensity with their yaw torque [40]. They can control the angular velocity of a panorama surrounding them not only by yaw torque but also by forward thrust, body posture or abdomen bending [82]. In ambiguous sensory situations, they actively switch between different perceptual hypotheses, they modify their expectations about the consequences of their actions by learning and they can actively shift their focus of attention restricting their behavioural responses to parts of the visual field [83,84]. These latest studies prompted further research into the process of the endogenous direction of selective attention in flies [85–89]. Martin Heisenberg realized early on [90] that such active processes entail the sort of fundamental freedom required for a modern concept of free will and keeps prominently advocating this insight today [91].

John Searle has described free will as the belief ‘that we could often have done otherwise than we in fact did’ [92]. Taylor & Dennett cite the maxim ‘I could have done otherwise’ [93]. Clearly, leeches and flies could and can behave differently in identical environments. While some argue that unpredictable (or random) choice does not qualify for their definition of free will [2], it is precisely the freedom from the chains of causality that most scholars see as a crucial prerequisite for free will. Importantly, this freedom is a necessary but not a sufficient component of free will. In order for this freedom to have any bearing on moral responsibility and culpability in humans, more than mere randomness is required. Surely, no one would hold a person responsible for any harm done by the random convulsions during an epileptic seizure. Probably because of such considerations, two-stage models of free will have been proposed already many decades ago, first by James [94], later also by Henri Poincaré, Arthur Holly Compton, Karl Popper, Henry Margenau, Daniel Dennett, Robert Kane, John Martin Fisher, Alfred Mele, Stephen Kosslyn, Bob Doyle and Martin Heisenberg (cited, reviewed and discussed in [7]), as well as Koch [9]: one stage generates behavioural options and the other one decides which of those actions will be initiated. Put simply, the first stage is ‘free’ and the second stage is ‘willed’. This implies that not all chance events in the brain must manifest themselves immediately in behaviour. Some may be eliminated by deterministic ‘selection’ processes before they can exert any effects. Analogous to mutation and selection in evolution, the biological process underlying free will can be conceptualized as a creative, spontaneous, indeterministic process followed by an adequately determined process, selecting from the options generated by the first process. Freedom arises from the creative and indeterministic generation of alternative possibilities, which present themselves to the will for evaluation and selection. The will is adequately determined by our reasons, desires and motives—by our character—but it is not *pre*-determined. John Locke (1689, p. 148) already separated free from ‘will’, by attributing free to the agent and not the will: ‘I think the question is not proper, whether the will be free, but whether a man be free’ [95]. Despite the long tradition of two-stage models of free will, only now are the first, tangible scientific pieces of evidence being published. For instance, the independent discovery of nonlinear mechanisms in brains from different phyla is compatible with such two-stage models [63,74]. Essentially, the existence of neural circuits implementing a two-stage model of free will ‘would mean that you can know everything about an organism's genes and environment yet still be unable to anticipate its caprices’ [96]. Importantly, this inability is not due to inevitable stochasticity beyond control; it is due to dedicated brain processes that have evolved to generate unpredictable, spontaneous actions in the face of pursuit–evasion contests, competition and problem-solving.

## 9. Consciousness and freedom

It thus is no coincidence that we all feel that we possess a certain degree of freedom of choice. It makes sense that depriving humans of such freedom is frequently used as punishment and the deprived do invariably perceive this limited freedom as undesirable. This experience of freedom is an important characteristic of what it is like to be human. It stems in part from our ability to behave variably. Voltaire expressed this intuition in saying ‘Liberty then is only and can be only the power to do what one will’. The concept that we can decide to behave differently even under identical circumstances underlies not only our justice systems. Electoral systems, our educational systems, parenting and basically all other social systems also presuppose behavioural variability and at least a certain degree of freedom of choice. Games and sports would be predictable and boring without our ability of constantly changing our behaviour in always the same settings.

The data reviewed above make clear that the special property of our brain that provides us with this freedom surely is independent of consciousness. Consciousness is not a necessary prerequisite for a scientific concept of free will. Clearly, a prisoner is regarded as un-free, irrespective of whether he is aware of it or not. John Austin [97] provides another instructive example ‘Consider the case where I miss a very short putt and kick myself because I could have holed it’. We sometimes have to work extremely hard to constrain our behavioural variability in order to behave as predictably as possible. Sports commentators often use ‘like a machine’ to describe very efficient athletes. Like practice, conscious efforts are able to control our freedom up to a certain degree. Compare, for instance, a line that you quickly drew on a piece of paper, with a line that was drawn with the conscious effort of making it as straight as possible. However, the neural principle underlying the process generating the variability is beyond total conscious control, requiring us to use rulers for perfectly straight lines. Therefore, the famous experiments of Benjamin Libet and others since then [2,4,5,98–100] only serve to cement the rejection of the metaphysical concept of free will and are not relevant for the concept proposed here. Conscious reflection, meditation or discussion may help with difficult decisions, but this is not even necessarily the case. The degree to which our conscious efforts can affect our decisions is therefore central to any discussion about the degree of responsibility our freedom entails, but not to the freedom itself.

## 10. The self and agency

In contrast to consciousness, an important part of a scientific concept of free will is the concept of ‘self’. It is important to realize that the organism generates an action itself, spontaneously. In chemistry, spontaneous reactions occur when there is a chemical imbalance. The system is said to be far from thermodynamic equilibrium. Biological organisms are constantly held far from equilibrium, they are considered open thermodynamic systems. However, in contrast to physical or chemical open systems, some of the spontaneous actions initiated by biological organisms help keep the organism away from equilibrium. Every action that promotes survival or acquires energy sustains the energy flow through the open system, prompting Georg Litsche to define biological organisms as a separate class of open systems (i.e. ‘subjects’; [101]). Because of this constant supply of energy, it should not be surprising to scientists that actions can be initiated spontaneously and need not be released by external stimuli. In controlled situations where there cannot be sufficient causes outside the organism to make the organism release the particular action, the brain initiates behaviour from within, potentially using a two-stage process as described above. The boy ceases to play and jumps up. This sort of impulsivity is a characteristic of children every parent can attest to. We do not describe the boy's action with ‘some hidden stimuli made him jump’—he jumped of his own accord. The jump has all the qualities of a beginning. The inference of agency in ourselves, others and even inanimate objects is a central component of how we think. Assigning agency requires a concept of self. How does a brain know what is self?

One striking characteristic of actions is that an animal normally does not respond to the sensory stimuli it causes by its own actions. The best examples are that it is difficult to tickle oneself and that we do not perceive the motion stimuli caused by our own eye saccades or the darkness caused by our eye blinks. The basic distinction between *self*-induced (re-afferent) and externally generated (ex-afferent) sensory stimuli has been formalized by von Holst & Mittelstaedt [102]. The two physiologists studied hoverflies walking on a platform surrounded by a cylinder with black and white vertical stripes. As long as the cylinder was not rotated, the animals seemed to behave as if they were oblivious to the stripes. However, as soon as the cylinder was switched on to rotate around the flies, the animals started to turn in register with the moving stripes, in an attempt to stabilize their orientation with respect to the panorama. Clearly, when the animals turned themselves, their eyes perceived the same motion stimuli as when the cylinder was rotated. The two scientists concluded that the animals detect which of these otherwise very similar motion signals are generated by the flies and which are not and dubbed this the ‘principle of reafference’. To test the possibility that the flies just blocked all visual input during self-initiated locomotion, the experimenters glued the heads of the animals rotated by 180° such that the positions of the left and right eye were exchanged and the proboscis pointed upwards. Whenever these ‘inverted’ animals started walking in the stationary striped cylinder, they ran in constant, uncontrollable circles, showing that they did perceive the *relative* motion of the surround. From this experiment, von Holst and Mittelstaedt concluded that self-generated turning comes with the expectation of a visual motion signal in the opposite direction that is perceived but normally does not elicit a response. If the visual motion signal is not caused by the animal, on the other hand, it most probably requires compensatory action, as this motion was not intended and hence not expected. The principle of reafference is the mechanism by which we realize which portion of the incoming sensory stream is under our own control and which portion is not. This is how we distinguish between those sensory stimuli that are consequences of our own actions and those that are not. Distinguishing self from ‘world’ is the prerequisite for the evolution of separate learning mechanisms for self- and world learning, respectively [43], which is the central principle of how brains balance actions and responses. The self/world distinction is thus the second important function of behavioural variability, besides making the organism harder to predict: by using the sensory feedback from our actions, we are constantly updating our model of how the environment responds to our actions. Animals and humans constantly ask: What happens if I do this? The experience of willing to do something and then successfully doing it is absolutely central to developing a sense of self and that we are in control (and not being controlled).

Thus, in order to understand actions, it is necessary to introduce the term self. The concept of self necessarily follows from the insight that animals and humans initiate behaviour by themselves. It would make no sense to assign a behaviour to an organism if any behavioural activity could, in principle, be traced back by a chain of causations to the origin of the universe. An animal or human being is the agent causing a behaviour, as long as no sufficient causes for this activity to occur are coming from outside the organism. Agency is assigned to entities who initiate actions themselves. Agency is crucial for moral responsibility. Behaviour can have good or bad consequences. It is the agent for whom the consequences matter the most and who can be held responsible for them.

## 11. Why still use the term free will today?

By providing empirical data from invertebrate model systems supporting a materialistic model of free will, I hope to at least start a thought process that abandoning the metaphysical concept of free will does not automatically entail that we are slaves of our genes and our environment, forced to always choose the same option when faced with the same situation. In fact, I am confident I have argued successfully that we would not exist if our brains were not able to make a different choice even in the face of identical circumstances and history. In this article, I suggest re-defining the familiar free will in scientific terms rather than giving it up, only because of the historical baggage all its connotations carry with them. One may argue that ‘volition’ would be a more suitable term, less fraught with baggage. However, the current connotations of volition as ‘willpower’ or the forceful, conscious decision to behave against certain motivations render it less useful and less general a term than free will. Finally, there may be a societal value in retaining free will as a valid concept, since encouraging a belief in determinism increases cheating [103]. I agree with the criticism that retention of the term may not be ideal, but in the absence of more suitable terms, free will; remains the best option.

I no longer agree that ‘ ‘‘free will’’ is (like ‘‘life’’ and ‘‘love’’) one of those culturally useful notions that become meaningless when we try to make them ‘‘scientific’’ ’ [96]. The scientific understanding of common concepts enrich our lives, they do not impoverish them, as some have argued [100]. This is why scientists have and will continue to try and understand these concepts scientifically or at least see where and how far such attempts will lead them. It is not uncommon in science to use common terms and later realize that the familiar, intuitive understanding of these terms may not be all that accurate. Initially, we thought atoms were indivisible. Today we do not know how far we can divide matter. Initially, we thought species were groups of organisms that could be distinguished from each other by anatomical traits. Today, biologists use a wide variety of species definitions. Initially, we thought free will was a metaphysical entity. Today, I am joining a growing list of colleagues who are suggesting it is a quantitative, biological trait, a natural product of physical laws and biological evolution, a function of brains, maybe their most important one.
